# Ultrafine brain intrinsic connectivity networks template via very-high-order independent component analysis of large-scale resting-state functional magnetic resonance imaging data

**DOI:** 10.3389/fnins.2025.1672129

**Published:** 2025-10-10

**Authors:** Shiva Mirzaeian, Kyle M. Jensen, Ram Ballem, Pablo Andrés Camazón, Jiayu Chen, Vince D. Calhoun, Armin Iraji

**Affiliations:** ^1^Tri-Institutional Center for Translational Research in Neuroimaging and Data Science (TReNDS), Atlanta, GA, United States; ^2^Department of Mathematics and Statistics, Georgia State University, Atlanta, GA, United States; ^3^Department of Psychology, Georgia State University, Atlanta, GA, United States; ^4^Department of Computer Science, Georgia State University, Atlanta, GA, United States; ^5^Institute of Psychiatry and Mental Health, Hospital General Universitario Gregorio Marañón, de Investigación Biomédica En Red de Salud Mental (CIBERSAM), Instituto de Salud Carlos III (ISCIII), School of Medicine, Universidad Complutense, Madrid, Spain; ^6^Neuroscience Institute, Georgia State University, Atlanta, GA, United States

**Keywords:** independent component analysis (ICA), resting-state fMRI (rsfMRI), granular intrinsic connectivity networks (ICNs), functional network connectivity (FNC), schizophrenia

## Abstract

Spatial group independent component analysis (sgr-ICA) is widely used in resting-state fMRI to identify intrinsic connectivity networks (ICNs). While lower-order decompositions reveal large-scale networks, higher-order models provide finer granularity but have been limited by small sample sizes. In this study, we applied sgr-ICA with 500 components to more than 100,000 subjects with rsfMRI to generate a robust fine-grained ICN template. Using this template, we examined whole brain functional network connectivity (FNC) in 502 individuals with schizophrenia and 640 typical controls and compared the findings with a lower order multiscale template. The 500-component template yielded a large set of reliable ICNs, particularly in the cerebellar and paralimbic regions, and revealed schizophrenia-related dysconnectivity patterns that were not detected at larger spatial scales. Specifically, we observed hypoconnectivity between the cerebellar and subcortical domains (basal ganglia and thalamus) and hyperconnectivity between the cerebellar domain and the visual, sensorimotor and higher cognitive domains. These results demonstrate that very high-order ICA can capture distinct fine-grained ICNs, improving the detection of disease-related connectivity differences and enriching current multiscale ICN templates. The derived ICNs can serve as a valuable reference for future studies and potentially enhance the clinical utility of rsfMRI in psychiatric research.

## 1 Introduction

### 1.1 High-order ICA and functional brain atlases

Models of brain communication in functional neuroimaging often rely on the assumption that temporal dependencies in neurophysiological data reflect functional connectivity (Friston, 2011). Independent component analysis (ICA), a widely used multivariate method, plays a crucial role in uncovering the functional organization of the brain (Calhoun et al., 2001b). ICA is a blind source separation technique that decomposes data into maximally independent components (Calhoun and Adali, 2012). Within neuroimaging research, it has proven valuable for identifying functional networks or intrinsic connectivity networks (ICNs) (Calhoun et al., 2001b). Two primary ICA-based approaches have been employed to estimate functional entities in the brain (Iraji et al., 2022b). The first involves performing ICA on individual subjects followed by clustering to identify shared patterns across samples (Esposito et al., 2005; Calhoun et al., 2001a). While this approach accounts for individual variability, it is susceptible to challenges like inter-individual differences, variations in data acquisition, and the dynamic nature of brain function. The second, more robust method involves a group-level ICA framework, which creates shared functional entities across a cohort and subsequently back-reconstructs these patterns for individual subjects (Calhoun et al., 2008). This approach has become a cornerstone for clinical and large-scale studies due to its reliability and consistency in capturing functional connectivity patterns across populations (Iraji et al., 2022b; Mirzaeian et al., 2024; Rashid et al., 2014).

Earlier studies employing group-level ICA focused on low-order models (20–45 components) that identified large-scale networks, such as default mode and salience networks (Beckmann et al., 2005; Calhoun et al., 2001b; Iraji et al., 2016). However, the inherent complexity of brain networks suggests the presence of smaller, functionally distinct subnetworks embedded within these large-scale systems (Kiviniemi et al., 2009a); hence, Higher order ICA (75–200 components) has been employed to identify such fine-grained ICNs, providing a more detailed representation of the brain's functional architecture (Mirzaeian et al., 2024; Abou Elseoud et al., 2009; Kiviniemi et al., 2009b; Allen et al., 2011; Iraji et al., 2022a). Although some studies have explored even higher model orders (e.g., 500 or 1,000 components), they are often applied to small datasets, highlighting the need for further extension to improve robustness and generalizability (Iraji et al., 2019).

The advent of population-level neuroimaging has introduced massive datasets with terabytes of high-resolution brain images, enabling researchers to uncover the neural underpinnings of individual differences (Iraji et al., 2022b). Large datasets improve the reliable estimation of ICNs and functional patterns by enhancing statistical power, reducing noise, and capturing more representative brain connectivity, which enables the derivation of robust, generalizable ICNs that better reflect individual and group differences. Such large-scale data requires efficient dimensionality reduction techniques to extract meaningful representations while managing computational challenges. Functional brain atlases, derived from these datasets, provide a structured framework to summarize complex connectivity patterns into image-derived phenotypes, which are critical for characterizing brain networks (Varoquaux and Craddock, 2013; Miller et al., 2016). Data-driven atlases from large datasets extracted from fMRI capture functional organization and individual variability and enhance reliability by improving statistical robustness and reducing noise (Jensen et al., 2024b). Among these, high-dimensional ICA-based atlases effectively delineate spatially continuous and functionally specific regions, making them valuable for studying brain connectivity.

We recently analyzed a large dataset of over 100,000 subjects using multi-model order ICA, ranging from 25 to 200 components, to develop the multiscale NeuroMark functional atlas of the brain (Iraji et al., 2022b). Subsequently, the functional atlas was refined, organized, and labeled, with each network described using terminology familiar to cognitive and affective neuroscience, resulting in NeuroMark-fMRI-2.2 (Jensen et al., 2024b). Building upon this foundation, we extend the ICA framework by implementing a group-level ICA at model order 500 on the same dataset to extract a replicable and reliable set of fine-grained ICNs. This extension results in a new template named NeuroMark-fMRI-500, which offers enhanced granularity and provides additional functional insights beyond those captured in NeuroMark 2.2.

### 1.2 Schizophrenia

Schizophrenia is a complex psychiatric disorder that impacts thinking, emotions, and daily functioning (Bowles, 2013). It is diagnosed based on a combination of symptoms, as there is no single test for the condition. The primary symptoms include positive symptoms such as hallucinations, delusions, and disorganized speech or behavior, and negative symptoms like lack of motivation, blunted affect, and emotional withdrawal, along with significant social difficulties (Bowles, 2013; Davies, 1988; Rekhi et al., 2019). Schizophrenia profoundly affects brain function by disrupting normal network connectivity, leading to patterns of hypo- and hyper-connectivity and reducing the brains ability to integrate and process information efficiently (Harikumar et al., 2023; Menon et al., 2022). For example, dysconnectivity between the motor cortices and cerebellar areas is a typical feature observed in schizophrenia (Walther et al., 2017). Additionally, dysfunction in the triple network, which includes the central executive network, the default mode network, and the salience network, has been implicated in the disorder, with their interactions often being deficient in schizophrenia (Menon, 2019, 2011; Mirzaeian et al., 2024). Although diverse methods, such as graph theory (Shahhosseini and Miranda, 2022), decomposition techniques, and seed-based analyses (Yu et al., 2012), have been used to study abnormal functional integration across brain circuits in schizophrenia, sgr-ICA has played a crucial role in identifying and characterizing schizophrenia-related patterns of functional connectivity. For instance, multiscale ICA, which investigates functional sources across multiple spatial scales, has uncovered sex-specific differences in schizophrenia (Iraji et al., 2022a). Similarly, Telescopic ICA, a novel method employing a recursive ICA strategy that leveraged information from larger ICNs to guide the extraction of smaller ICNs, revealed significant associations in the posterior cortex and precuneus regions linked to auditory hallucinations in individuals with schizophrenia (Mirzaeian et al., 2024). Using higher orders in ICA, which involves analyzing a large number of fine-grained components, allows for the identification of more subtle and complex brain network interactions, shedding light on changes in functional connectivity associated with schizophrenia (Iraji et al., 2022b, 2019).

In this study, we analyze functional network connectivity (FNC) from 1,142 subjects, including typical controls (TC) and individuals with schizophrenia (SZ), extracted using NeuroMark-500 and its association with cognitive scores. Our results show that NeuroMark-500 captures a relatively large number of ICNs within the cerebellar area, revealing their strong hypoconnectivity with subcortical-extended thalamic regions as well as subcortical-basal ganglia regions, and hyperconnectivity with the sensorimotor cortex. These findings underscore the cerebellums significant role in brain function and its disruption in schizophrenia.

## 2 Materials and equipment

### 2.1 Data collection and data preparation

For this study, we relied on the same dataset and quality control (QC) procedures as described in Iraji et al. (2022b), Jensen et al. (2024b), and Du et al. (2020), without introducing any modifications. These works explicitly address inter-site variability and ensure consistent preprocessing and quality control across all included data. Resting-state fMRI (rsfMRI) datasets were used from 100,517 subjects, sourced from over twenty private and public datasets. A complete list of these datasets, along with details on accessing additional information, is provided in Supplementary Section 1 in Iraji et al. (2022b). These datasets originate from cohorts with varying sex and diagnosis ratios, age distributions, and imaging protocols that differ in spatial and temporal resolution. The QC criteria included (a) a minimum of 120 time points (volumes) in the rsfMRI time series, (b) mean framewise displacement less than 0.25 mm, (c) head motion transitions within 3° rotation and 3 mm translation in any direction, (d) high-quality registration to an echo-planar imaging template, and (e) spatial overlap between individual masks and the group mask, including the top and bottom ten slices, exceeding 80%. These criteria were chosen for their feasibility across diverse datasets (Iraji et al., 2022b). Applying these established QC criteria resulted in 57,709 individuals (57.4 %) passing the QC requirements, forming the QC-passed dataset. Data preprocessing follows the procedures described in Iraji et al. (2022b), and Du et al. (2020). When available, preprocessed data from a given dataset were used; otherwise, preprocessing pipelines were applied. The preprocessing steps included rigid body motion correction, slice timing correction, and distortion correction, using the FMRIB Software Library (FSL v6.0, https://fsl.fmrib.ox.ac.uk/fsl/fslwiki/) and the Statistical Parametric Mapping (SPM12, https://www.fil.ion.ucl.ac.uk/spm/) toolboxes within the MATLAB environment. Next, preprocessed subject data were warped into the Montreal Neurological Institute (MNI) space using an echo-planar imaging (EPI) template. This approach has been shown to outperform structural templates (Calhoun et al., 2017) when distortion correction is unavailable or unfeasible, which was the case for this study. Finally, subject data were resampled to 3 mm^3^ isotropic voxels and spatially smoothed using a Gaussian kernel with a 6 mm full width at half-maximum (FWHM).

## 3 Method

### 3.1 Independent component analysis

We performed spatial Group-level ICA (sgr-ICA) using the Group ICA Toolbox (GIFT) (Calhoun et al., 2001b; Iraji et al., 2020) on data. The steps for sgr-ICA are as follows. First, we applied variance normalization on voxel time courses and conducted subject spatial principal components analysis (PCA) to retain the principal components (PCs) with subject-level variance exceeding 95%. Next, we performed group spatial PCA by concatenating subject PCs to further reduce the dimensionality of the data and decrease the computational demands of sgr-ICA (Calhoun et al., 2008). We used a memory-efficient subsampled time PCA (STP) approach due to data size to calculate Group PCs (Rachakonda et al., 2016). Next, we ran sgr-ICA using the Infomax algorithm (Bell and Sejnowski, 1995) with model order 500, resulting in 500 fine scale components.

### 3.2 Identifying replicable ICNs: construction of the NeuroMark-500 template

We used 500 independent components to identify the set of replicable fine-grained ICNs as NeuroMark-500 template. First, we assessed the reliability and quality of the components using the ICASSO index quality (IQ) (Himberg et al., 2004). Components with IQ value below 0.80 were also excluded from further analysis. Secondly, we evaluated the replicability of the components using a split-half approach. We randomly split the QC-passed data into two independent halves and applied sgr-ICA separately on each half, resulting in 500 components per half. To identify the best-matching components between the two halves, each 3D component image was first masked to include only brain voxels and then reshaped into a one-dimensional vector. Pearson correlation was computed between these vectors, using the full sample as a reference, to quantify spatial similarity. This procedure was repeated 50 times, and components with an average similarity below 0.80 (Himberg et al., 2004) across the 50 iterations were excluded.

**Figure 1:**
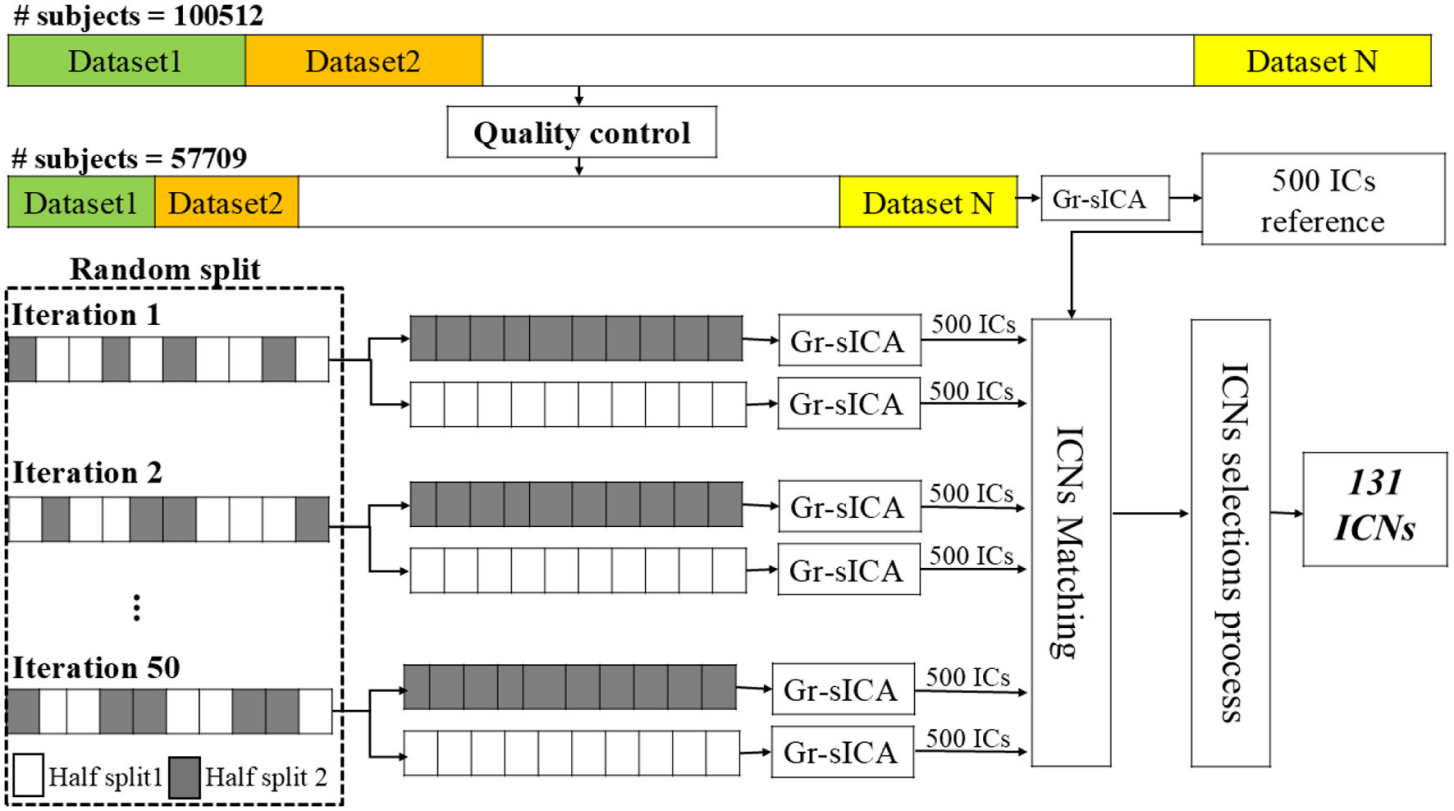
Analysis pipeline: Data from 100,512 subjects underwent preprocessing and quality control (QC), resulting in 57,709 subjects passing QC. Spatial Group-level Independent Component Analysis (sgr-ICA) was then applied to generate 500 independent components (ICs). The QC-passed dataset was randomly split into two halves, and sgr-ICA was applied to each half to independently generate 500 ICs. This process was repeated independently 50 times. Next, through component matching and selection, 131 stable and reliable ICs were identified and labeled as intrinsic connectivity networks (ICNs), forming the NeuroMark-500 template.

By applying these two criteria we obtained a subset of components that were replicable and reliable. Finally, to classify components as ICNs, we applied the same procedure in Du et al. (2020) and Iraji et al. (2022b), selecting components that exhibited high spatial overlap with gray matter and low spatial similarity to motion artifacts, ventricular signals, or other known artifact patterns. For components located in cortical regions, we additionally ensured that their peak activation was within gray matter. Subcortical components, being smaller and often surrounded by white matter or cerebrospinal fluid (CSF), were instead evaluated using a general criterion requiring that they be predominantly located within gray matter to avoid missing reliable components. Based on these, 131 ICNs were identified as NeuroMark-fMRI-order-500 template. The identified ICNs are categorized into domain and subdomain labels according to NeuroMark 2.2 template (Jensen et al., 2024b) using spatial similarity measured by Pearson correlation. The overall workflow of the proposed method is illustrated in Figure 1.

After establishing these replicable ICNs, we leveraged them to study their application in FNC and group comparisons between TC and SZ.

### 3.3 Clinical applications: functional connectivity alterations (HC vs. SZ), reliability assessments, cognitive associations

We utilized the BSNIP dataset (Tamminga et al., 2013; Clementz et al., 2021) for further analysis, which comprises 1,142 subjects, including TC and SZ. Participants underwent the Structured Clinical Interview for the Diagnostic and Statistical Manual of Mental Disorders IV (DSM-IV), and assessments were conducted while participants were clinically stable.

To estimate subject-specific independent components and time courses, we employed multivariate-objective optimization ICA with reference (MOO-ICAR), using the NeuroMark-500 components derived from sgr-ICA as spatial priors. The group-level sgr-ICA provides a robust and replicable template of functional networks across the population, ensuring stability and consistency. MOO-ICAR leverages this template to extract subject-specific components while minimizing artifacts, effectively capturing individual variability (Du and Fan, 2012). This two-step strategy balances group-level reliability with subject-level specificity and is computationally efficient for large datasets, making it more practical than alternatives such as Independent Vector Analysis with Gaussian-Laplacian (IVA-GL). We then applied time course cleaning procedures, including detrending and despiking, to eliminate drifts, sudden fluctuations, and residual artifacts (Braun et al., 2011; Yaesoubi et al., 2015). Finally, we computed static FNC (sFNC) matrices by calculating pairwise correlations between the cleaned time courses of different brain domains across the entire dataset (Jafri et al., 2008; Allen et al., 2011).

To statistically assess group differences in sFNC between TC and SZ, first, we applied a linear regression model to regress out potential confounding factors, including age, sex, scanning site, and mean framewise displacement. Next, we conducted two-sample t-tests on the cleaned sFNC to compare the TC and SZ groups.

To evaluate the reliability and stability of the group comparison results, we performed two complementary analyses. First, we randomly split the data into two non-overlapping subsets and independently conducted regression and group comparison analyses on each subset, resulting in two t-statistic maps. We then computed the Pearson correlation between these maps. This process was repeated for 20 iterations to assess the reliability of the detected group differences across different random data partitions. Second, to evaluate the stability of the findings, we randomly sampled 80% of the dataset and performed regression and group comparison analyses to generate a t-statistic map. We then computed the Pearson correlation between this map and the one derived from the full dataset. This procedure was also repeated for 20 iterations, providing a measure of the consistency of the group comparison results across random subsamples.

Additionally, we evaluated the relationships between cleaned sFNC and cognitive performance measures using Pearson correlation coefficient. We removed group effects by demeaned both cleaned sFNC and cognitive scores to ensure that the observed associations reflect relationship between variables rather than being skewed by group-level variations. Cognitive performance was measured using standardized z-scores from the Brief Assessment of Cognition in Schizophrenia (BACS) (Keefe et al., 2004). This analysis enabled us to explore how functional connectivity differences relate to distinct cognitive domains.

### 3.4 ICN comparison: NeuroMark-500 vs. NeuroMark 2.2

We compared ICNs from the NeuroMark-500 with ICNs from NeuroMark 2.2. NeuroMark 2.2 is a reliable and replicable multi-spatial-scale ICNs template using sgr-ICA with order ranging from 25 to 200. We performed comparisons based on spatial map size, component distribution across functional domains, and the ability to capture group differences. ICN size was defined by the number of voxels exhibiting z-scored intensity values greater than 1.96 (Z > 1.96). To evaluate the ability to capture group differences, we extracted subject-specific time courses of NeuroMark 2.2 ICNs from the same dataset used in our group comparison analysis and applied an identical methodological pipeline to both approaches, ensuring a consistent and direct comparison.

## 4 Results

### 4.1 ICN results

**Figure 2:**
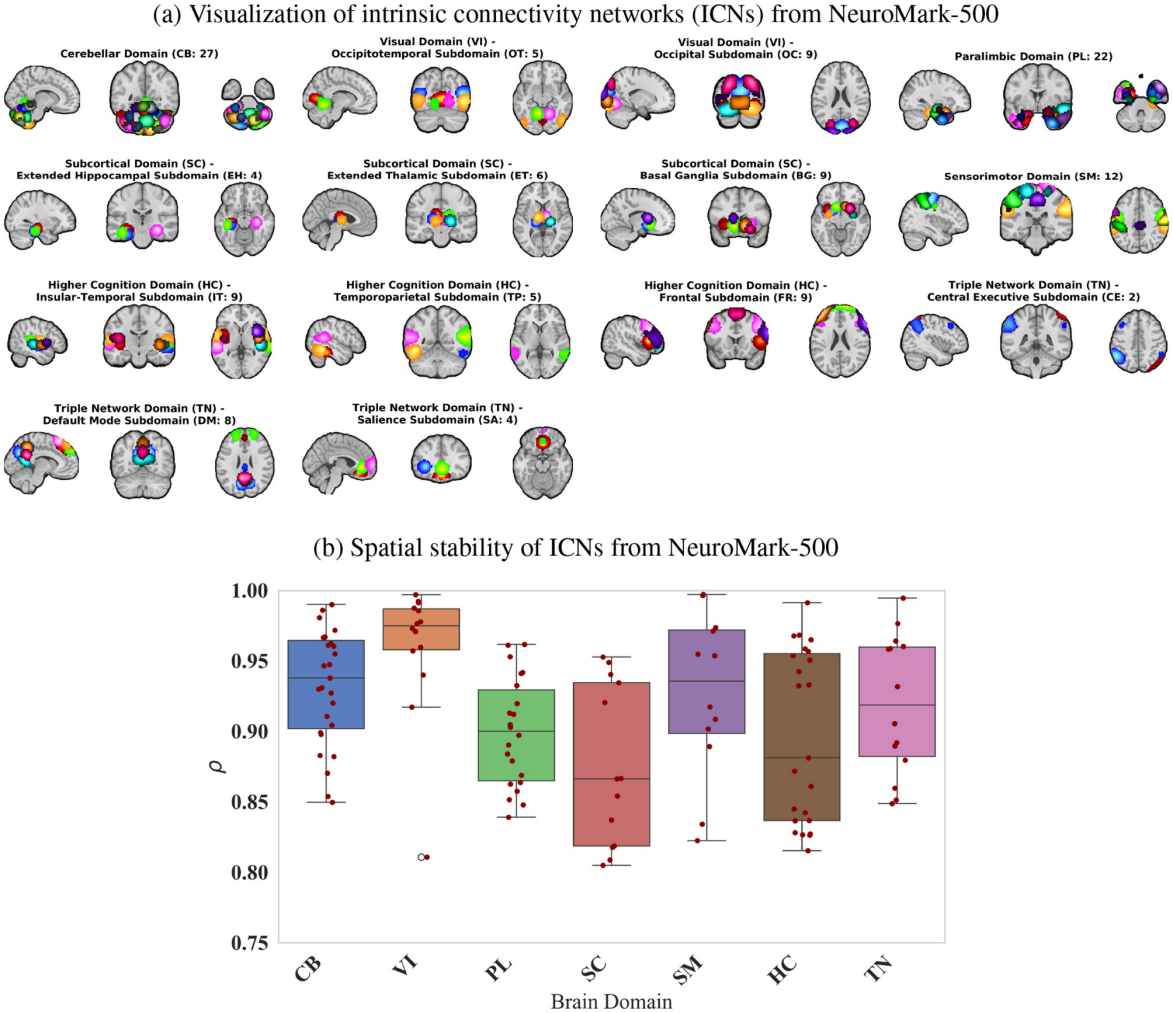
**(a)** Representation of 131 spatial maps of ICNs from NeuroMark-500 for each of the seven domains and 14 subdomains- labeling based on based on NeuroMark 2.2 template: cerebellar (CB), visual-occipitotemporal (VI-OT), visual-occipital (VI-OC), paralimbic (PL), subcortical extended hippocampal (SC-EH), subcortical-extended thalamic (SC-ET), subcortical-basal ganglia (SC-BG), sensorimotor (SM), higher cognition-insular temporal (TC-IT), higher cognition-temporoparietal (HC-TP), higher cognition-frontal (HC-FR), triple network-central executive (TN-CE), triple network-default mode (TN-DM), and triple network-salience (TN-SA). The spatial maps of ICs are thresholded with a z-score of 1.96 (*p*-value < 0.05). **(b)** Spatial stability of the 131 ICNs from NeuroMark-500, evaluated across 50 split-half iterations.

Based on the criteria outlined in Section 3.2, we identified 131 reliable ICNs extracted from NeuroMark-500. Figure 2a presents the spatial maps of these ICNs, organized into domains and subdomains according to the NeuroMark 2.2 template. Among these, 27 ICNs were classified within the cerebellar domain. The higher cognitive and paralimbic domains also exhibited substantial contributions, with 23 and 22 ICNs, respectively.

Figure 2b presents the stability index, which quantifies the spatial similarity of each ICN generated from 2 half split across 50 iterations. This metric provides insight into the consistency of ICN identification and reflects the robustness of the analysis. The results indicate that the stability index for all ICNs exceeds 0.80, demonstrating high reproducibility. ICNs within the visual domain exhibited the highest stability (>0.95), followed by those in the cerebellar, sensorimotor, and triple-network domains, which also showed strong stability.

### 4.2 Clinical application results

**Figure 3:**
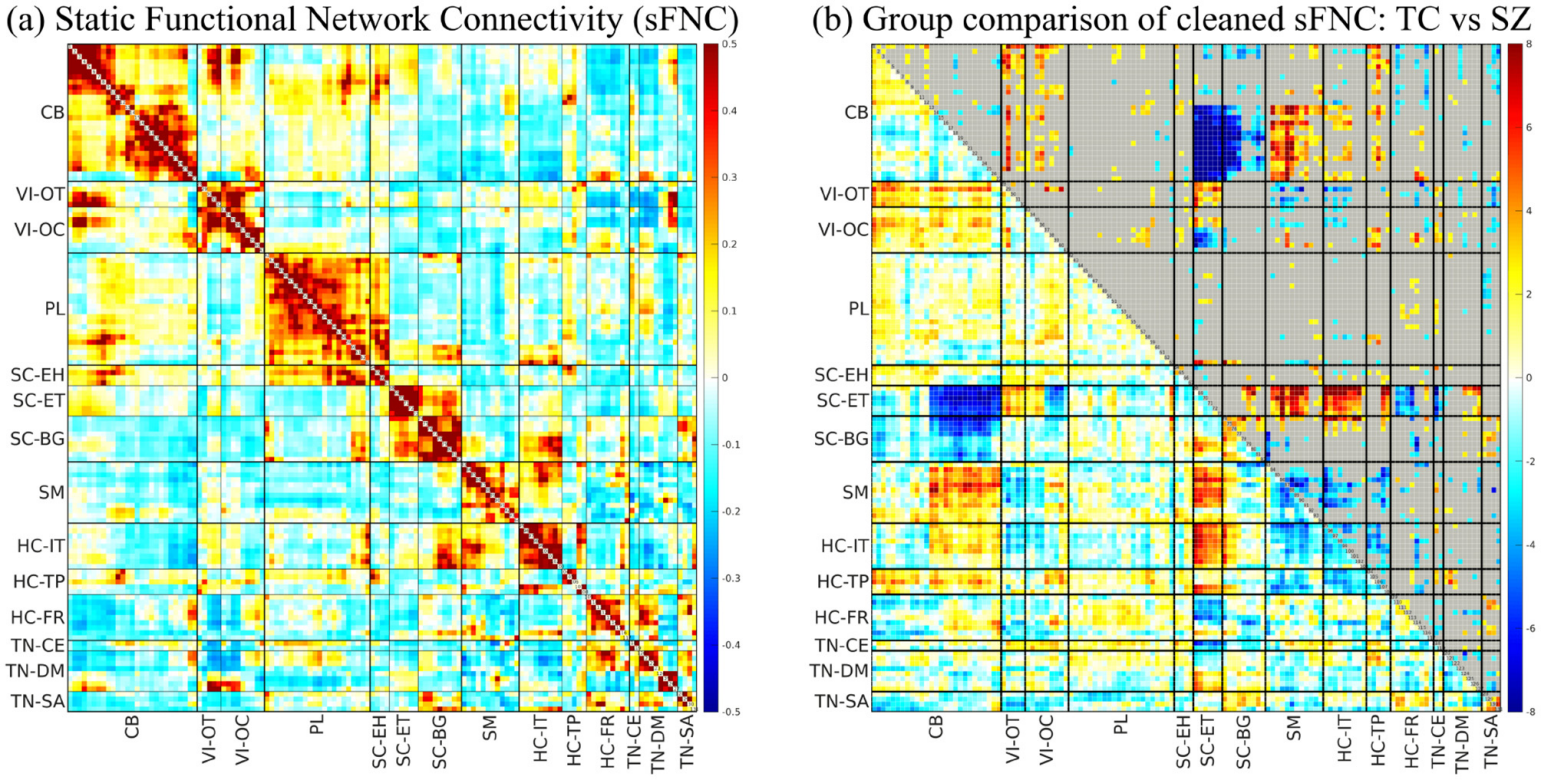
Clinical application results of ICNs from NeuroMark-500 for 1,142 subjects in the BSNIP dataset. **(a)** Heatmap of sFNC between ICNs, showing averaged pairwise correlations between time courses of different brain domains. **(b)** Group comparison of cleaned sFNC (TC vs. SZ) using NeuroMark-500: The lower triangle represents −log(p-value) × sign(t-value), while the upper triangle highlights connectivity with p-values < 0.05. P-values are FDR corrected. Blue indicates hypoconnectivity in the SZ, while red represents hyperconnectivity in the SZ. Labels: cerebellar (CB), visual-occipitotemporal (VI-OT), visual-occipital (VI-OC), paralimbic (PL), subcortical extended hippocampal (SC-EH), subcortical-extended thalamic (SC-ET), subcortical-basal ganglia (SC-BG), sensorimotor (SM), higher cognition-insular temporal (HC-IT), higher cognition-temporoparietal (HC-TP), higher cognition-frontal (HC-FR), triple network-central executive (TN-CE), triple network-default mode (TN-DM), and triple network-salience (TN-SA).

Figure 3 presents the sFNC matrix and the group comparison analysis between cohorts using the NeuroMark-500 template from the BSNIP sample. The sFNC heatmap displays pairwise correlations between ICN time courses, providing a detailed view of functional connectivity patterns across all subjects. The accompanying group comparison plot illustrates the cleaned sFNC differences between TC and SZ, based on the statistical approach described in Section 3.3. The group comparison analysis reveals both hyperconnectivity and hypoconnectivity patterns in the SZ group relative to TC. Notably, there is pronounced hypoconnectivity between the cerebellar domain and both the subcortical-extended thalamic and subcortical-basal ganglia regions. Conversely, the SZ group exhibits significant hyperconnectivity between the cerebellar domain and sensorimotor, insular-temporal, and temporoparietal sub-domains. Additionally, the thalamic region shows significant increased connectivity with sensorimotor, insular-temporal, and temporoparietal areas, highlighting widespread alterations in cross-sub-domain functional integration.

To evaluate the robustness of the group comparison analysis, we assessed both stability and reliability. The stability analysis, performed by comparing t-statistic maps derived from random 80% subsets against the full dataset for 20 iterations, yielded a mean Pearson correlation of 0.97, indicating highly consistent results across sub-samples. The reliability analysis, based on comparing t-statistic maps from two non-overlapping halves of the dataset across 20 iterations, resulted in a mean correlation of 0.80, demonstrating high reproducibility of the detected group differences.

**Figure 4:**
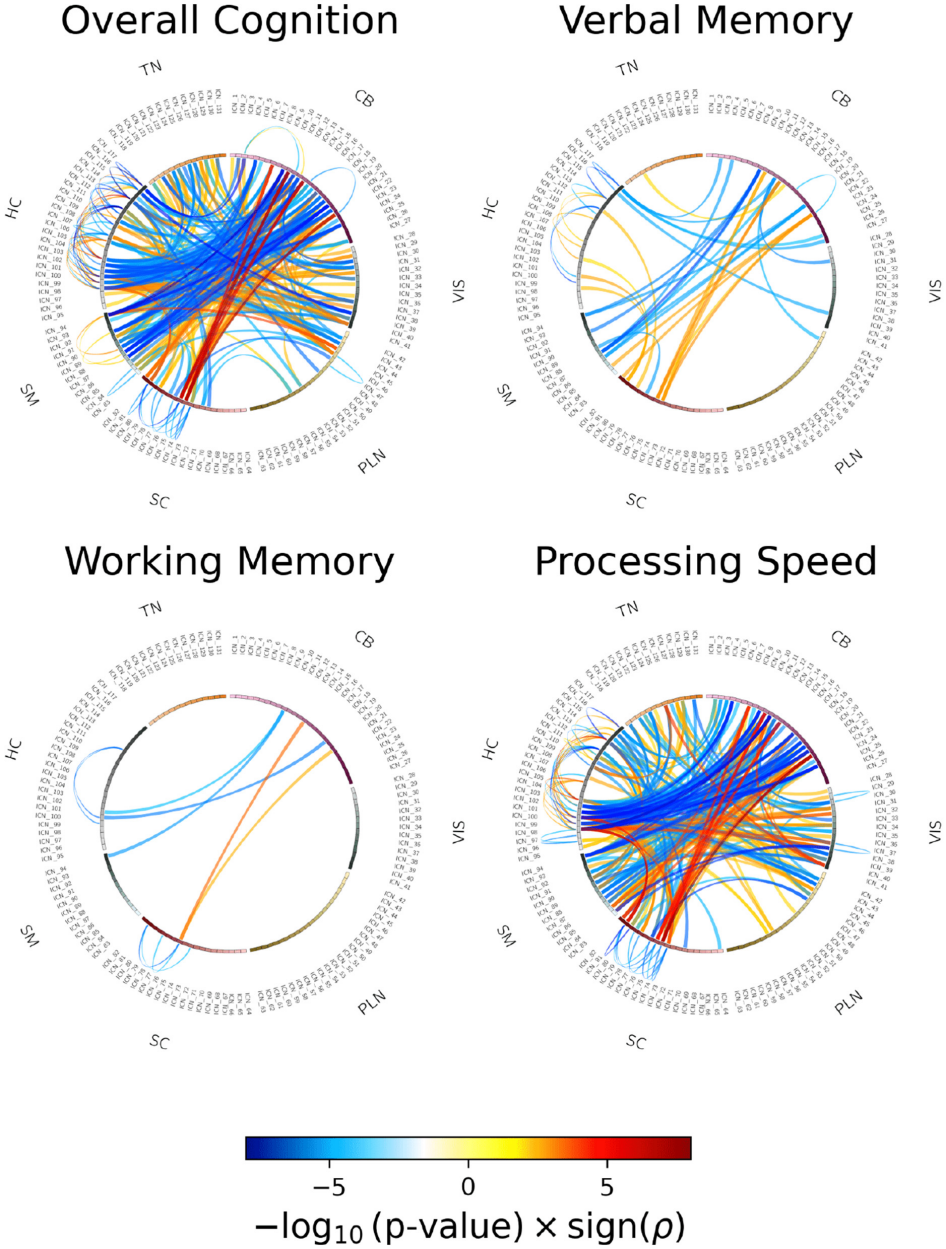
Associations between cognitive scores and cleaned sFNC from NeuroMark-500. The connectograms highlight Pearson correlation coefficients between cleaned sFNC and cognitive measures including overall cognition, verbal memory, working memory, and processing speed—showing significant correlations (*p* < 0.005). *p*-values are FDR corrected.

Figure 4 illustrates the correlations between cleaned sFNC and cognitive performance after regressing out diagnostic effects from both variables. The connectograms display highly significant Pearson correlations (*p* < 0.005), with p-values corrected for multiple comparisons using the false discovery rate (FDR) method. Cognitive measures include overall cognition, verbal memory, working memory, and processing speed. The correlation analysis shows a generally consistent pattern for all cognitive associations. The plots exhibit a significant positive association between sFNC in subcortical-extended hippocampal and subcortical-basal ganglia with cerebellar domain, indicating that strong connectivity in these regions supports cognitive function. Conversely, cerebellar-sensorimotor, thalamic-sensorimotor and thalamic-insular temporal, and thalamic-frontal is negatively correlated with the same cognitive measures, suggesting that increasing connectivity between these networks may be linked to cognitive impairments.

### 4.3 Comparison of ICNs from NeuroMark-500 vs. NeuroMark 2.2

**Figure 5:**
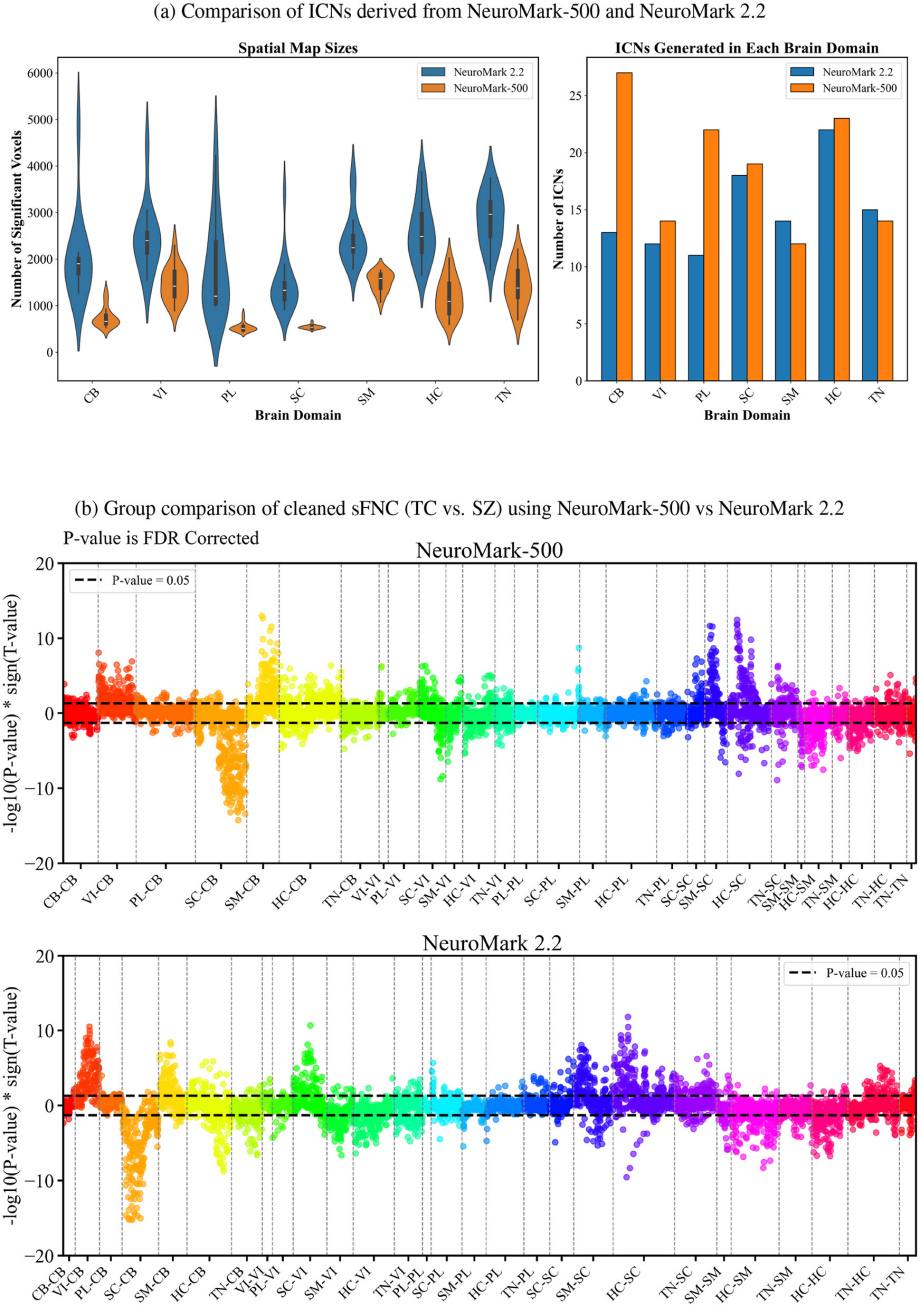
Comparison of ICNs derived from NeuroMark-500 and NeuroMark 2.2. **(a)** Spatial map sizes and ICNs distribution across brain domains. **(b)** Group comparison of sFNC (TC vs. SZ) using NeuroMark-500 vs. NeuroMark 2.2: Positive side indicates hypoconnectivity in the SZ, while negative side represents hyperconnectivity in the SZ.

Figure 5a compares the ICNs generated using the NeuroMark-500 with ICNs produced by NeuroMark 2.2, focusing on the number of ICNs identified within each brain domain and ICN size. The analysis reveals that ICNs derived from the NeuroMark-500 are in general smaller in size across all brain domains. Additionally, NeuroMark-500 approach identifies a greater number of ICNs across cerebellar, visual, paralimbic, subcortical and higher cognitive domain. This combination of smaller ICN sizes and an increased number of ICNs highlights the NeuroMark-500 capacity to achieve finer granularity in brain network parcellation.

Figure 5b compares the group comparison results between TC and SZ from NeuroMark-500 vs. NeuroMark 2.2. The plot shows that the two approaches exhibit a generally consistent pattern, which provides additional support for the validity and interpretability of the results obtained using NeuroMark-500 framework. However, notable differences are observed in the paralimbic and cerebellar domains, where the results extracted from NeuroMark-500 reveals stronger and more widespread diagnostic effects—particularly in connections between the cerebellar and sensorimotor domains, and between the paralimbic and cerebellar domains. The results also indicate that three brain domains—cerebellar, subcortical, and sensorimotor—demonstrate diagnostic associations with multiple other brain domains. Specifically, the cerebellar domain shows significant hypoconnectivity with the subcortical domain, higher cognitive networks, and the triple network. Additionally, a significant hypoconnectivity is observed between the higher cognitive and sensorimotor domains. On the other hand, significant hyperconnectivity involving the cerebellar domain is observed with the visual, sensorimotor, and higher cognitive domains. The sensorimotor domain also exhibits significant hyperconnectivity with the subcortical domain.

**Figure 6:**
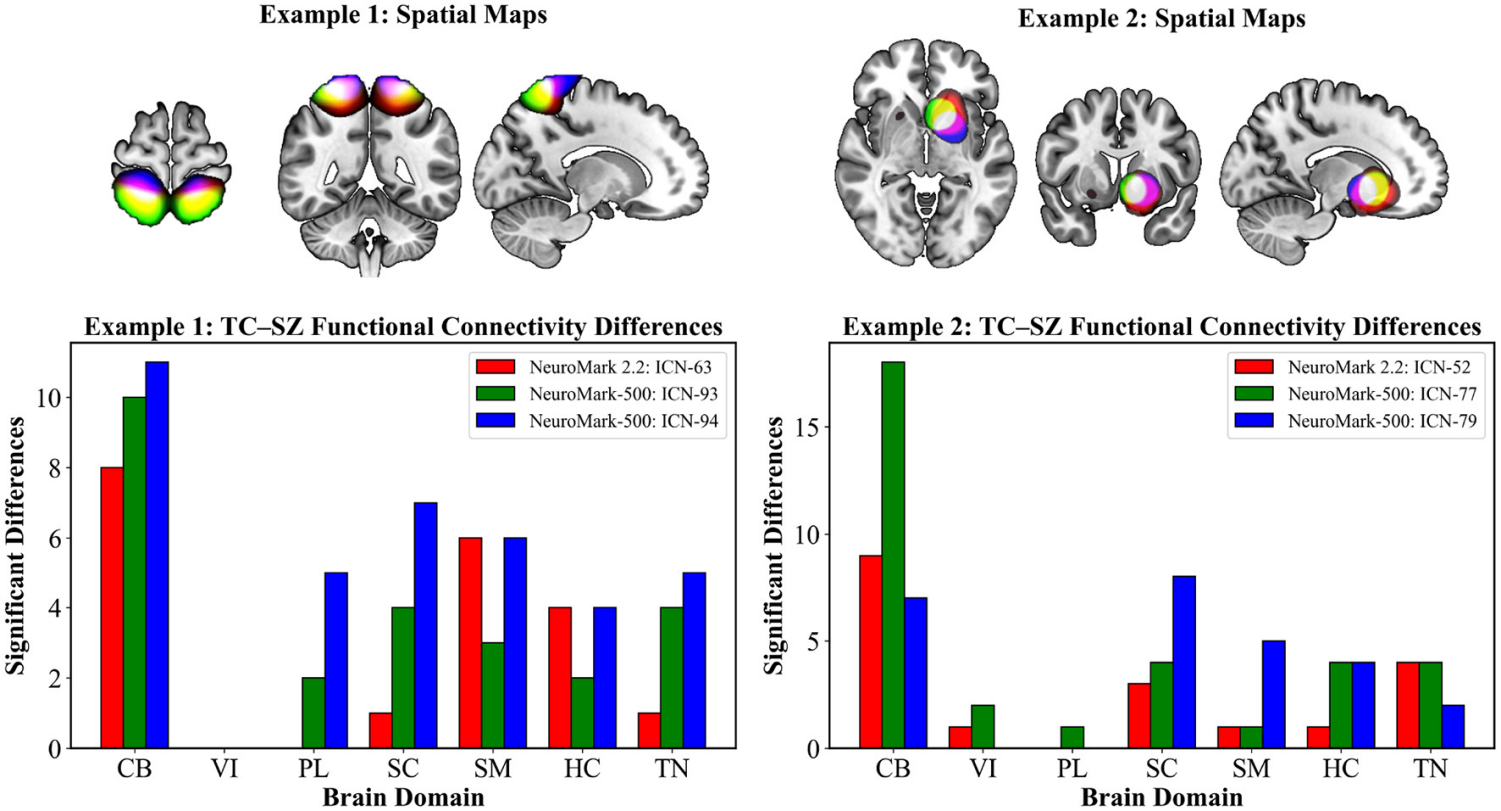
Barplot illustrates the number of significant functional network connections showing group differences between TC and SZ from the group comparison analysis. Each example compares an ICN from NeuroMark 2.2 with its two most spatially similar ICNs derived from NeuroMark-500. The NeuroMark-500 decomposition reveals finer-grained ICNs and identifies additional significant group differences highlighting its enhanced sensitivity for capturing clinically relevant connectivity alterations. Red represents spatial maps from NeuroMark 2.2, blue and green show spatial maps from NeuroMark-500, yellow is overlap between red and green, magenta is overlap between red and blue, white is overlap between all three.

Figure 6 presents two representative examples highlighting the capability of NeuroMark-500 to capture finer-grained brain networks and provide more detailed insights into the functional patterns of the SZ group, compared to the larger scale ICNs used in NeuroMark 2.2. In each example, we selected one ICN from NeuroMark 2.2 and identified its correspondence into two distinct ICNs from NeuroMark-500 using Pearson correlation of spatial maps.

In Example 1, the sensorimotor ICN (ICN 63) from NeuroMark 2.2 showed spatial correlations of 0.73 and 0.55 with two sensorimotor ICNs (ICN 93 and ICN 94, respectively) from NeuroMark-500. In Example 2, the subcortical–basal ganglia ICN (ICN 52) from NeuroMark 2.2 exhibited the highest spatial similarity with ICNs 77 and 79 from NeuroMark-500, with correlation coefficients of 0.61 and 0.59, respectively. These spatial maps visually and quantitatively confirm that increasing the ICA model order allows a single ICN to branch out into multiple more localized components, thereby revealing more detailed functional brain organization. We also examined diagnosis association between cohorts across the corresponding ICNs. The NeuroMark-500 revealed additional significant diagnostic associations that were not captured by NeuroMark 2.2. In both examples, ICNs derived from NeuroMark-500 capture significant diagnostic effects within the paralimbic domain that are not detected using the NeuroMark 2.2 template. Additionally, the number of connectivity pairs showing significant group differences is higher when using the finer-grained ICNs from NeuroMark-500 compared to the coarser, large-scale ICNs, highlighting the benefits of increased spatial resolution in detecting subtle group effects.

## 5 Discussion

This study demonstrates the efficacy of higher order ICA for resting-state fMRI data, identifying 131 ICNs with high granularity. The high reproducibility of ICNs, as evidenced by a stability index exceeding 0.80 across 50 runs, underscores the robustness of this method. Our findings highlight the prominent involvement of the cerebellum, with 27 out of 131 networks spatially overlapping with this region. Although, historically understudied in neuroimaging research, emerging studies have established the cerebellum as a major hub in the brain's functional circuitry (Kawabata et al., 2022; Schmahmann, 2018; Dong et al., 2022), more recently, subregions in the cerebellum have been established as having specialized roles in integrating and processing information (Kawabata et al., 2022; Schmahmann, 2018; Dong et al., 2022). The relatively large number of ICNs we identified within the cerebellum underscores its significant role in brain function and lends further support toward future research aiming to explore the functional contributions of the cerebellum in cognition.

Beyond the cerebellum, our results also reveal a notable enrichment of ICNs within the paralimbic domain, with 22 ICNs classified under this category. The paralimbic domain, centered around regions adjacent to limbic structures—including the entorhinal cortex, medial temporal lobe, and temporal pole—is known to support a wide range of higher-order cognitive functions such as emotion regulation, memory, language, and learning (Juarez et al., 2013; Kiehl, 2006). The substantial number of ICNs identified within the paralimbic domain emphasizes its critical role in overall brain function and further motivates future investigations into its specific contributions to cognition. Crucially, our group comparison analysis in Figure 6 revealed significant differences between TC and SZ cohort specifically within the paralimbic and subcortical domains—differences that were not detected using the NeuroMark 2.2 template. This finding highlights the added value of higher order ICA, which enables the extraction of more focal ICNs, thereby increasing sensitivity to subtle alterations in functional connectivity.

Additionally, our comparison of sFNC between TC and SZ using NeuroMark 2.2 and NeuroMark-500 as more fine scale ICNs reveals that, while the overall pattern remains similar, a key distinction arises in the hippocampus subdomain. The NeuroMark-500 appears to enhance the separation of the hippocampus from neighboring regions. In contrast, the larger ICNs may capture effects influenced by adjacent structures, particularly the thalamus. This suggests that increased granularity may offer a more precise delineation of functional networks, which could be critical for accurately characterizing the role of the hippocampus in psychopathology-related connectivity alterations.

Moreover, our results reveal strong hypoconnectivity within subcortical regions and the cerebellar, along with hyperconnectivity in sensorimotor-cerebellar areas. These connectivity disruptions align with prior studies reporting similar dysconnectivity patterns in schizophrenia (Jensen et al., 2024a; Hwang et al., 2021; Yan et al., 2023), reinforcing the critical role of subcortical and cerebellar dysfunction in the disorder. This underscores the need for more targeted investigations into these regions and their contributions to schizophrenia-related network alterations.

Our findings further suggest that schizophrenia-related cognitive deficits are closely linked to altered subcortical and cerebellar connectivity. The observed hypoconnectivity within thalamic, basal ganglia, and cerebellar may indicate impaired integration of these key subcortical structures with the rest of the brain, potentially disrupting executive function, attention, and motor coordination. Given that these regions play a vital role in cognitive control and memory, their reduced connectivity may contribute to widespread cognitive impairments observed in schizophrenia (Mana et al., 2024). Moreover, the positive correlation between subcortical-cerebellar connectivity and cognitive scores suggests that stronger functional interactions between these areas benefit cognitive performance. This finding aligns with previous research emphasizing the role of the thalamus and basal ganglia in cognitive regulation (Barch and Ceaser, 2011; Silver and Feldman, 2005). Conversely, hyperconnectivity between CB and SM networks was negatively correlated with cognitive function, which may reflect a compensatory but inefficient mechanism (Mana et al., 2024). Excessive sensorimotor-cerebellar engagement may interfere with higher cognitive functions rather than enhance them, mirroring prior findings linking disrupted CB-SM interactions to cognitive deficits in schizophrenia (Schmahmann, 2018). Overall, these results reinforce the idea that schizophrenia is characterized by widespread dysconnectivity across subcortical, cerebellar, and sensorimotor networks. Distinct patterns of hypo- and hyperconnectivity appear to play a crucial role in cognitive dysfunction, highlighting the complex interplay between brain network disruptions and schizophrenia-related cognitive deficits.

This study opens several avenues for future research and has some important limitations to consider. First, NeuroMark-500 is based on a single high–model-order decomposition, which enables the extraction of a large number of fine-grained ICNs but does not yet leverage a multi–model-order framework. In contrast, the NeuroMark 2.2 template utilizes a multi–model-order ICA strategy, combining components derived from different lower model orders. There is significant promise in developing a unified network template that merges these two approaches. Such a combined template could capitalize on the strengths of both—capturing large-scale, robust networks as well as subtle, focal ICNs—offering a more comprehensive and flexible tool for mapping brain functional architecture in both typical and clinical populations. Second, while this work demonstrates the feasibility of a model order of 500, future research should explore even higher model orders to further refine network granularity, provided that sufficiently large datasets and rigorous validation procedures are employed to ensure robustness and reproducibility. Third, the observed modular organization within the sFNC matrix involving the cerebellar domain suggests the possible existence of sub-domains within this region. Future studies should examine this structure in greater depth and aim to classify the cerebellar domain into distinct, functionally meaningful sub-domains. Moreover, our analyses are limited to static FNC, which assumes that connectivity patterns remain constant throughout the scan. Because brain connectivity is inherently dynamic, temporal fluctuations may provide additional insights into schizophrenia-related network alterations. Incorporating dynamic FNC analyses could help capture time-varying connectivity patterns and better characterize transient network states. Finally, although higher-order ICA appears effective in detecting subtle connectivity differences, it remains unclear how well these patterns predict clinical outcomes or cognitive impairments in schizophrenia. Validation with larger, independent, and longitudinal datasets will be necessary to establish the clinical relevance and translational value of these findings.

## 6 Conclusion

This study demonstrates the power of fine-grained ICNs from large resting-state fMRI datasets in understanding brain functions. By applying a 500-component ICA model to an extensive dataset of over 58,000 individuals, we achieved 131 highly reproducible and spatially refined ICNs of the brain and introduced NeuroMark-500. Furthermore, our analysis of sFNC in schizophrenia revealed distinct patterns of hypo- and hyperconnectivity, particularly within subcortical, cerebellar, and sensorimotor regions, highlighting their critical role in schizophrenia-related cognitive deficits. These findings suggest that NeuroMark-500 can serve as a valuable template for future neuroimaging studies, with potential clinical applications in psychiatric research.

## Data Availability

The original contributions presented in the study are included in the article/supplementary material, further inquiries can be directed to the corresponding author.
